# HCN Channel Modulation of Synaptic Integration in GABAergic Interneurons in Malformed Rat Neocortex

**DOI:** 10.3389/fncel.2017.00109

**Published:** 2017-04-19

**Authors:** Asher J. Albertson, Andrew S. Bohannon, John J. Hablitz

**Affiliations:** Department of Neurobiology, University of Alabama at BirminghamBirmingham, AL, USA

**Keywords:** inhibitory interneurons, *I*_h_, synaptic integration, cortical dysplasia, neocortex

## Abstract

Cortical malformations are often associated with pharmaco-resistant epilepsy. Alterations in hyperpolarization-activated, cyclic nucleotide-gated, non-specific cation (HCN) channels have been shown to contribute to malformation associated hyperexcitability. We have recently demonstrated that expression of HCN channels and *I*_h_ current amplitudes are reduced in layer (L) 5 pyramidal neurons of rats with freeze lesion induced malformations. These changes were associated with an increased EPSP temporal summation. Here, we examine the effects of HCN channel inhibition on synaptic responses in fast spiking, presumptive basket cells and accommodating, presumptive Martinotti, GABAergic interneurons in slices from freeze lesioned animals. In control animals, fast spiking cells showed small sag responses which were reduced by the HCN channel antagonist ZD7288. Fast spiking cells in lesioned animals showed absent or reduced sag responses. The amplitude of single evoked EPSPs in fast spiking cells in the control group was not affected by HCN channel inhibition with ZD7288. EPSP ratios during short stimulus trains at 25 Hz were not significantly different between control and lesion groups. ZD7288 produced an increase in EPSP ratios in the control but not lesion groups. Under voltage clamp conditions, ZD7288 did not affect EPSC ratios. In the control group, accommodating interneurons showed robust sag responses which were significantly reduced by ZD7288. HCN channel inhibition increased EPSP ratios and area in controls but not the lesioned group. The results indicate that HCN channels differentially modulate EPSPs in different classes of GABAergic interneurons and that this control is reduced in malformed rat neocortex.

## Introduction

Synaptic excitation of L5 pyramidal cells is regulated by hyperpolarization-activated, cyclic nucleotide-gated (HCN) channels (Berger et al., 2001; Berger and Lüscher, 2003). These channels mediate *I*_h_, a non-inactivating, cationic current activated by membrane hyperpolarization (Robinson and Siegelbaum, 2003). *I*_h_ can be active at resting membrane potentials, resulting in reduced neuronal input resistance and cell depolarization (Magee, 1998). Inhibition of *I*_h_ enhances intrinsic neuronal excitability (Magee, 1998), increases the time-course of distally evoked EPSPs and boosts temporal summations of EPSPs (Williams and Stuart, 2000; Berger et al., 2001), allowing somatic membrane potentials to move significantly closer to threshold (Berger et al., 2001). The influence of *I*_h_ on intrinsic excitability and synaptic integration in GABAergic interneurons is less well understood.

Excitability in neocortical circuits is controlled by GABAergic interneurons (Burkhalter, 2008; Marín, 2012; Le Magueresse and Monyer, 2013). Specific classes of GABAergic cells target defined regions of pyramidal cells (Kawaguchi and Kubota, 1997). The soma and proximal dendrites are contacted by parvalbumin-expressing, non-accommodating, fast-spiking, basket cells (Kawaguchi and Kubota, 1997). Fast-spiking basket cells contribute to generation of rhythmic network activity (Buhl et al., 1998; Tamás et al., 2000) and modulate pyramidal cell firing (Somogyi et al., 1998). Alterations in basket cell excitability have also been implicated in a variety of neurological disorders including epilepsy (Powell et al., 2003; Marín, 2012). In contrast, somatostatin (SOM)-expressing Martinotti cells are identified by ascending axon collaterals which reach L1 and ramify extensively (Kawaguchi and Kubota, 1997), can be sub-classified as regular spiking or burst firing (Wang et al., 2004; Uematsu et al., 2008), display spike frequency accommodation, mediate frequency-dependent disynaptic inhibition (Silberberg and Markram, 2007) and target oblique, apical and tuft dendrites of L5 pyramidal cells (Berger et al., 2010).

The apical dendrites of L5 pyramidal cells stain prominently with HCN antibodies (Lörincz et al., 2002; Notomi and Shigemoto, 2004), a finding which has fostered close examination of the function of HCN channels in intrinsic and synaptic excitability in these cells. Neocortical inhibitory interneurons do not typically show HCN staining (Lörincz et al., 2002; Notomi and Shigemoto, 2004), although HCN4 immunoreactivity has been observed in parvalbumin-expressing cells in the hippocampal CA1 region (Hughes et al., 2013). Current clamp electrophysiological studies, however, have demonstrated the presence of membrane potential “sag” responses in certain classes of interneurons, suggesting the presence of *I*_h_ currents. Martinotti cells display a “sag” back towards baseline during a hyperpolarizing current pulse and a “rebound” overshoot upon repolarization (Ma et al., 2006), responses typically mediated by HCN channels. Sag potentials have not been widely investigated in neocortical neurogliaform cells or basket cells but do not appear to be prominent (Kawaguchi, 1995; Oláh et al., 2009).

Due to the role of HCN channels in decreasing cellular excitability, alterations in *I*_h_ and HCN channel expression have been studied in many models of epilepsy. The kainate (Shin et al., 2008) and pilocarpine (Jung et al., 2007) models of temporal lobe epilepsy as well as the amygdala kindling model (Powell et al., 2008) all are associated with reductions in HCN channels. HCN1 knockout animals have reduced seizure thresholds (Huang et al., 2009), whereas HCN2 knockout animals exhibit an absence epilepsy phenotype (Ludwig et al., 2003). Furthermore, excised tissue from human epilepsy patients has reduced *I*_h_ currents (Wierschke et al., 2010). Cortical dysplasia is associated with severe pharmacoresistant epilepsy (Leventer et al., 2008; Guerreiro, 2009). Regions of cortical dysplasia in epilepsy patients are often removed in hopes of reducing seizures, though this has variable efficacy (Sisodiya, 2000; Krsek et al., 2009). The reason for the epileptogenic nature of dysplastic tissue in cortical malformations is poorly understood. The tissue is characterized by intrinsic hyperexcitability, abnormal cell types, and gliosis (Guerreiro, 2009). Features of human cortical dysplasia, including dyslamination and gliosis, are reproduced in rats with cortical freeze-lesions (Dvořàk et al., 1978). We have shown that rat L5 pyramidal neurons near a freeze-induced microgyrus have reduced *I*_h_ currents and show increases in intrinsic and synaptic excitability (Albertson et al., 2011). In the present article, we test the hypothesis that alterations in HCN channel function alters synaptic integration and passive properties of GABAergic interneurons in rats with freeze lesions.

## Materials and Methods

### Ethics Statement

All experiments were performed in accordance with the National Institutes of Health *Guide for the Care and Use of Laboratory Animals* with protocols approved by the University of Alabama at Birmingham Institutional Animal Care and Use Committee. We made every effort to minimize pain and discomfort. Focal freeze lesions were induced in postnatal day 1 rats as previously described (Albertson et al., 2011). In brief, newborn rat pups were anesthetized by hypothermia, and a small incision was made in the scalp. A 2 mm copper rod cooled to approximately −50°C was placed on the surface of the skull for 3 s. After the scalp was sutured, the animals were warmed and returned to their home cages. Rats were allowed to recover for a minimum of 20 days before recordings were made.

#### Preparation of Acute Neocortical Brain Slices

Rats were anesthetized with isoflurane and decapitated. The brain was quickly removed and placed in ice-cold oxygenated (95% O_2_/5% CO_2_, pH 7.4) cutting solution consisting of (in mM): 135 N-Methyl-D-glucamine, 1.5 KCl, 1.5 KH_2_PO_4_, 23 choline HCO_3_, 0.4 ascorbic acid, 0.5 CaCl_2_, 3.5 MgCl_2_ and 25 D-glucose (Tanaka et al., 2008). A vibratome (Microm, Waldorf, Germany) was used to cut 300 μM thick coronal brain slices. Slices were obtained from an area of cortex containing the microgyrus in freeze lesioned animals, and a corresponding region in control animals. The slices were maintained for 40–60 min at 37°C in oxygenated recording solution containing (in mM) 124 NaCl, 2.5 KCl, 10 D-glucose, 26 NaHCO_3_, 2.0 Ca^2+^, 2.0 Mg^2+^ and then kept at room temperature. For recording, individual slices were transferred to a recording chamber and continuously perfused (4 ml/min) with oxygenated recording solution kept at 32 ± 1°C.

#### Whole Cell Recording

Neurons were visualized with either a Leica DM LFSA (Leica Microsystems Wetzlar GMBH, Wetzlar, Germany) microscope equipped with Nomarski optics or a Zeiss Axio Examiner D1 (Carl Zeiss Inc., Thornwood, NY, USA) microscope equipped with Dodt contrast optics, each equipped with a 40×-water immersion lens and infrared illumination. L5 interneurons were identified by their somatic shape, size, absence of a prominent apical dendrite, distance from the pial surface, intrinsic properties and spiking properties.

Whole cell recordings were obtained from visually and physiologically identified cells in L5 of the neocortex. Signals were acquired with either an Axopatch 200B amplifier (Molecular Devices LLC, Sunnyvale, CA, USA) or a ELC-03XS npi bridge balance amplifier (npi Electronic GmbH, Tamm, Germany) controlled by Clampex software via a Digidata 1322A or 1550A interface (Molecular Devices). Responses were digitized at 5 kHz, and analyzed offline with Clampfit software. Patch electrodes with an open tip resistance of 2–5 MΩ were pulled from borosilicate glass tubes. Tight seals (>1 GΩ) were obtained between the patch electrodes and the neurons before using suction to break into whole cell mode. Only recordings with a series resistance <25 MΩ were used for the study and recordings in which a >20% increase in series resistance was observed were excluded. Patch electrodes were filled with an internal solution containing (in MM): 125 K-gluconate, 10 KCl, 10 HEPES, 2 Mg-ATP, 0.2 Na-GTP, 0.5 EGTA, and had an adjusted pH and osmolarity of 7.3 and 290, respectively. GABA_A_ receptors were inhibited with bicuculline methiodide (BIC; 10 μM; Sigma Aldrich, St. Louis, MO, USA) in all experiments. Synaptic responses were evoked with a concentric bipolar stimulating electrode (FHC, Bowdoin, ME, USA) or twisted pair nichrome bipolar electrode positioned within several hundred micrometers of the recorded interneuron. Single EPSPs were evoked at a rate of 0.1 Hz with 100 μs pulses 20–200 μA in amplitude. Trains of five EPSPs at 25 Hz were used to examine synaptic integration. Traces shown are the average of 5–10 consecutive responses.

#### Data Analysis

Data is either shown as dots representing individual data points or as means ± the standard error. Statistical analysis was carried out with either a Students *t*-test, using paired *t*-tests for analysis of data acquired before and after drug application, or with a two-way ANOVA. Student’s *t*-tests were one-tailed unless otherwise noted. *P* < 0.05 was considered significant.

Sag responses were evaluated using hyperpolarizing current injection in 50 pA intervals. Sag amplitude was calculated as the maximum membrane hyperpolarization following current onset minus the steady state potential prior to offset of the hyperpolarizing current injection. Synaptic integration was evaluated using EPSP ratios calculated as the percent change in the amplitude of the fifth evoked event relative to the amplitude of the first event. The total area under the curve of evoked trains was normalized to the amplitude of the first EPSP to account for potential changes in input as stimulus intensity was kept constant for pre- and post-drug trials.

#### Drugs

Drugs were stored in frozen stock solution and dissolved in the recording solution prior to each experiment. BIC was present at all times in all conditions. After recording control responses, ZD7288 (20 μM; Tocris Bioscience, Ellisville, MO, USA) was washed in for 10 min to block HCN channels.

## Results

### HCN Channel Mediated Responses in Fast-Spiking GABAergic Interneurons in Malformed Cortex

GABAergic interneurons serve to regulate network activity (Cobb et al., 1995; Pouille and Scanziani, 2001). Changes to their intrinsic membrane or synaptic integration properties could influence circuit behavior. Moreover, differential effects of alterations in HCN channels on inhibitory vs. excitatory cell types might contribute to epileptic pathology. Our previous work has shown that HCN mediated responses in L5 pyramidal cells are reduced in animals with freeze lesions (Albertson et al., 2011). We therefore tested the hypothesis that, if present, HCN mediated responses would also be reduced in L5 GABAergic interneurons in rats with freeze lesions.

**Figure 1:**
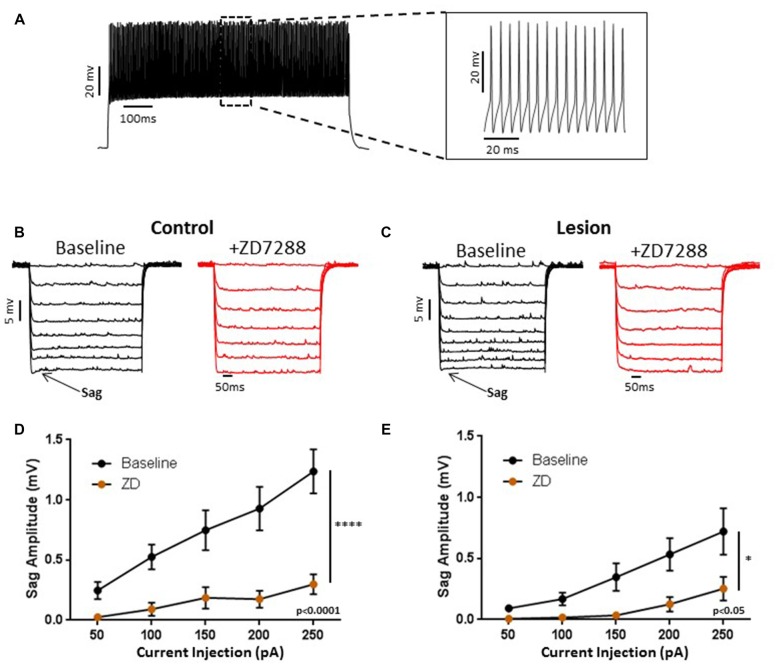
**Characterization of hyperpolarization-activated “sag” responses in fast-spiking interneurons in control and lesion groups. (A)** Typical spiking response in a fast spiking L5 interneuron from a control animal. Insert shows spikes at higher time resolution. Similar responses were seen in the lesion group. **(B)** Typical voltage “sag” (arrow) in response to hyperpolarizing current pulses from control animals before (black trace) and after (red trace) administration of ZD7288. **(C)** Characterization of voltage sag responses as in **(B)** for fast-spiking interneurons from control animals. Error bars are mean ± SEM. ZD7288 significantly reduced sag response amplitude (*n* = 22, 2-way ANOVA, *p* < 0.0001). **(D)** Specimen records of responses to hyperpolarizing current pulses in the lesioned group. Typical voltage “sag” (arrow) in response to hyperpolarizing current pulses from lesioned animals before (black trace) and after (red trace) administration of ZD7288 are shown. **(E)** Plot of voltage dependent “sag” as a function of injected current amplitude in interneurons from lesioned animals. ZD7288 significantly reduced the sag amplitude in lesioned animals (*n* = 16, 2-way ANOVA, *p* < 0.05). *Indicates *p* < 0.05; ****p < 0.0001.

We obtained somatic, whole-cell patch clamp recordings from L5 interneurons in neocortical slices from control and lesioned animals. Recordings in lesioned animals were obtained in the hyperexcitable zone 1–2 mm lateral to the lesion (Jacobs et al., 1996; Hablitz and DeFazio, 1998). Neurons were identified as fast spiking, presumed basket, cells based on firing properties. As shown in Figure 1A, depolarizing current pulses evoked high frequency, non-accommodating spike trains in the recorded cells; action potentials on an expanded time base are shown in the insert in Figure 1A. These cells had input resistances close to 200 MΩ, narrow spike widths and prominent fast AHPs, as described previously for basket cells (Kawaguchi, 2001). In pyramidal neurons, negative current pulses result in a hyperpolarization of the membrane potential followed by “sag” of the membrane potential back towards rest. This is caused by activation of the HCN mediated inward *I*_h_ current (Berger et al., 2001). Upon pulse offset, there is a rebound depolarization due to HCN channel closure (Berger et al., 2001; Albertson et al., 2011). We observed small sag responses in L5 basket cells from control animals upon sufficiently strong hyperpolarization (Figure 1B, left). These responses had an atypical time course rapidly reaching a peak and declining rapidly. These sag responses were not accompanied by a rebound depolarization. To test for possible mediation by HCN channels, the HCN channel antagonist ZD7288 was bath applied (20 uM, 10 min). This treatment greatly reduced sag responses (Figure 1B, right). Figure 1C shows a plot of sag amplitude as a function of injected current amplitude; ZD7288 significantly reduced sag response amplitude (*n* = 22, 2-way ANOVA, *p* < 0.0001). The responses to hyperpolarizing current injection before and after bath application of ZD7288 for a L5 basket cell in the hyperexcitable zone of a lesioned animal are shown in Figure 1D. ZD7288 significantly reduced the sag amplitude in lesioned animals (Figure 1E; *n* = 16, 2-way ANOVA, *p* < 0.05). Overall, the sag responses to a 250 pA negative current step were significantly reduced in interneurons from freeze lesioned rats compared to controls (Control: 1.2 ± 0.2 mV, *n* = 22; Lesion: 0.72 ± 0.2 mV, *n* = 16; *t-test*, *p* < 0.05). Rebound responses were virtually absent in both groups (Control: 0.14 mV ± 0.1, *n* = 10; Lesion: 0.32 mV ± 0.2, *n* = 7; data not shown). These results are consistent with decreased HCN channel functioning in L5 fast spiking cells in lesioned animals.

**Figure 2:**
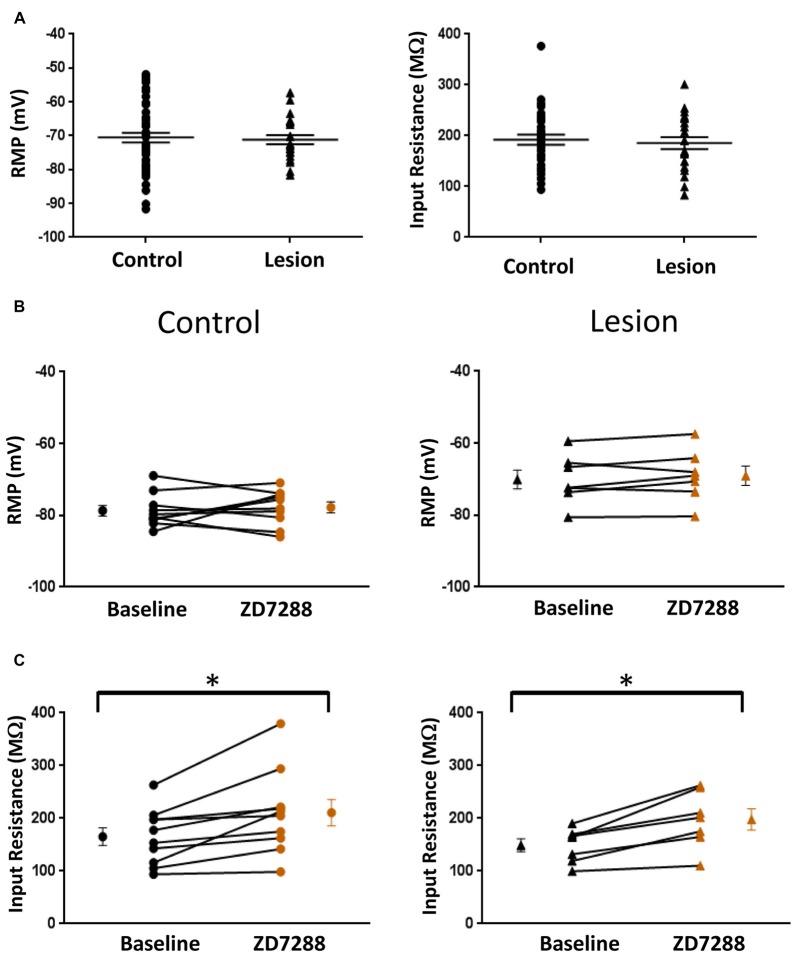
**Comparison of fast-spiking interneuron intrinsic properties in control and freeze lesion groups. (A)** Histogram of RMP (left) and input resistance (right) results from fast spiking interneurons in control and lesioned animals. Membrane potentials (Control: *n* = 50, Lesion: *n* = 22) and input resistances (Control: *n* = 35, Lesion: *n* = 22) were not significantly different. **(B)** Application of ZD7288 to inhibit HCN channels did not significantly alter the RMP of cells from control (left; *n* = 10) or lesioned (right; *n* = 7) animals. **(C)** The input resistance of cells from both control (left; *n* = 10) and lesioned (right; *n* = 7) animals increased following ZD7288 application. *p < 0.05, paired *t-test*. Error bars are mean ± SEM.

The resting membrane potential (Control: −70.55 ± 1.4 mV, *n* = 50; Lesion: −71.16 ± 1.4 mV, *n* = 22; two-tailed *t-test*, *p* = 0.79) and input resistance (Control: 192.2 ± 9.9 MΩ, *n* = 35; Lesion: 185.5 ± 11.8 MΩ, *n* = 22; two-tailed *t-test*, *p* = 0.67) of a population of interneurons from control and lesioned animals were not significantly different (Figure 2A, left and right, respectively). HCN channel inhibition did not significantly affect the RMP of neurons from the control (Baseline: −78.7 ± 1.5 mV, *n* = 10; ZD7288: −77.8 ± 1.5 mV, *n* = 10; paired *t-test*, *p* = 0.29) or lesioned group (Baseline: −70.1 ± 2.6 mV, *n* = 7; ZD7288: −69.1 ± 2.7 mV, *n* = 7; paired *t-test*, *p* = 0.13; Figure 2B; left and right, respectively), but input resistance was increased in the presence of ZD7288 in both control (Baseline: 165.3 ± 16.8 MΩ, *n* = 10; ZD7288: 210.9 ± 25.1 MΩ; *n* = 10; paired *t-test*, *p* < 0.01) and lesioned groups (Baseline: 148.8 ± 12.2 MΩ, *n* = 7; ZD7288: 197.7 ± 20.3 MΩ, *n* = 7; paired *t-test*, *p* < 0.01; Figure 2C).

### Effect of HCN Channel Inhibition on Evoked EPSP/EPSCs and EPSP Ratios in L5 Fast Spiking Interneurons

**Figure 3:**
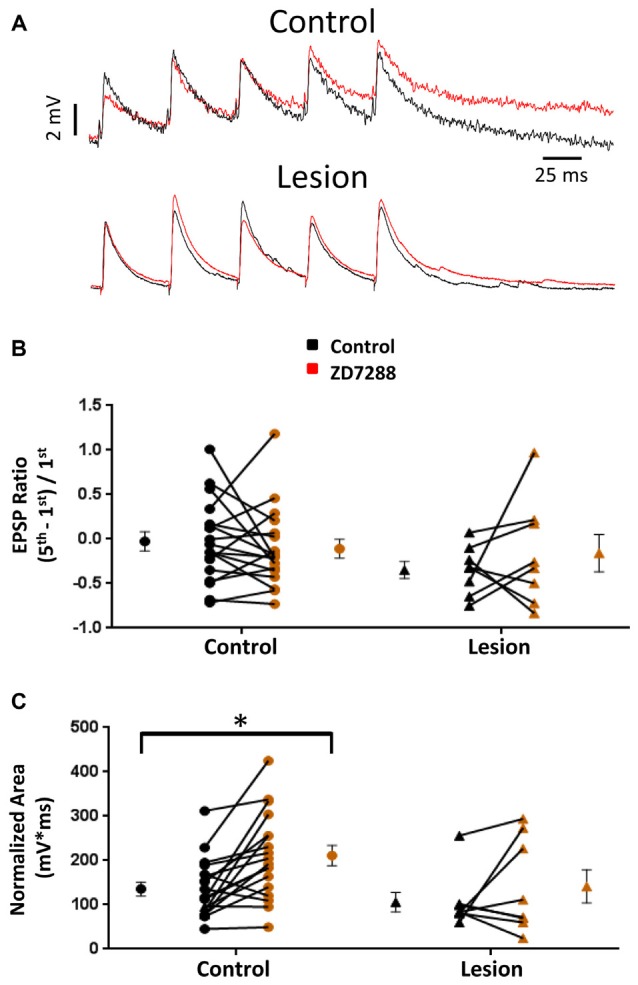
**Effects of HCN channel inhibition on EPSPs in L5 fast spiking interneurons. (A)** Specimen records of trains of EPSPs evoked at 25 Hz before (black) and after (red) application of ZD7288 in control (top) and lesioned (bottom) animals. **(B)** HCN channel inhibition did not alter EPSP ratios in control or freeze lesioned animals. No significant difference was seen in pre-drug ratios between control and freeze lesioned rats. **(C)** HCN channel inhibition significantly increased the EPSP area in control but not freeze lesioned rats. No difference was seen in pre-drug EPSP area between control and freeze lesioned rats. Control: *n* = 18, Lesion: *n* = 8; *p < 0.05, paired *t-test*. Error bars are mean ± SEM.

HCN channel inhibition has been reported to increase EPSP amplitude and temporal summation in L5 pyramidal neurons (Berger et al., 2001). We tested the hypothesis that, given the presence of only a small sag response in L5 basket cells, evoked EPSPs would not be significantly altered following HCN channel inhibition. In the presence of bicuculline (10 μM) to block GABAergic inhibition, synaptic responses were evoked with a stimulating electrode placed adjacent to the recorded cell. In order to evoke similar amplitude responses in control and lesioned groups, the threshold current necessary to consistently evoke an EPSP was determined for each cell. Responses were then obtained before and after bath application of the HCN channel antagonist ZD7288 (20 uM, 10 min). No significant effect of HCN channel inhibition on first EPSP amplitude was observed (Figure 3A, upper; Baseline: 4.3 ± 0.4 mV, *n* = 21; ZD7288: 3.8 ± 0.4 mV, *n* = 21; paired *t*-test, *p* = 0.07). Similar responses from a fast spiking neuron in the lesion group are shown in Figure 3A, lower. First EPSP amplitude was not significantly increased in the presence of ZD7288 (Baseline: 3.8 ± 0.6 mV, *n* = 13; ZD7288: 2.4 ± 0.5 mV, *n* = 13; paired *t-test*, *p* > 0.05).

HCN channel expression along the long apical dendrites of pyramidal neurons and Purkinje cells filters dendritic EPSPs and limits synaptic integration (Magee, 1999; Angelo et al., 2007). Loss of HCN channels in models of epilepsy significantly increases EPSP summation (Shin et al., 2008) allowing synaptic inputs to more effectively drive excitatory neurons toward action potential threshold. A similar enhancement of EPSP summation associated with HCN channel loss was observed in pyramidal neurons in malformed cortex (Albertson et al., 2011). Increased effectiveness of synaptic input may contribute to the hyperexcitability observed in epilepsy. However, if loss of HCN channels similarly increased effectiveness of EPSP summation in inhibitory interneurons, their increased output could offset the excitatory changes in pyramidal neurons. We therefore determined the effects of HCN channel inhibition on repetitive synaptic activation in interneurons in slices from control and lesioned animals. To examine this, we evoked five EPSPs at 25 Hz in L5 fast spiking cells. Figure 3A, upper, shows representative synaptic responses before and during ZD7288 application. Trains of 25 Hz stimulation in control animals resulted in variable amounts of EPSP integration. Temporal integration was quantified by determining a ratio comparing the peak amplitude of the first and fifth response [(EPSP5-EPSP1)/EPSP1] (Magee, 1999). In the presence of ZD7288 to inhibit HCN channels, no significant increase in EPSP ratios was observed in the control group (Control: −0.03 ± 0.11, *n* = 18; ZD7288: −0.11 ± 0.11, *n* = 18; paired *t*-test, *p* = 0.24; Figure 3A, upper traces) as shown in Figure 3B. Examining the results from the lesioned group also indicated that ZD7288 had no significant effect on EPSP ratios (Control: −0.35 ± 0.10, *n* = 8; ZD7288: −0.16 ± 0.21, *n* = 8; paired *t-test*, *p* = 0.21; Figure 3B, right). Baseline ratios did not differ between control and lesion groups (Control: −0.03 ± 0.11, *n* = 18; Lesion: −0.35 ± 0.10, *n* = 8; two-tailed *t-test*, *p* = 0.08). This is consistent with our observation of minimal sag responses in controls and virtual absence of such responses in the lesion group. It has been reported that HCN channel inhibition also results in an increase in EPSP area (Magee, 1998; Berger et al., 2001). When we quantified the area of evoked EPSP trains, it was found that ZD7288 significantly increased the area in the control group (Control: 135.5 ± 15.6 mV*ms, *n* = 18; ZD7288: 211.2 ± 22.7 mV*ms, *n* = 18; paired *t-test*, *p* < 0.05; Figure 3C) but did not significantly change the EPSP area in the lesion group (Control: 105.9 ± 21.9 mV*ms, *n* = 8; ZD7288: 141.5 ± 37.6 mV*ms, *n* = 8; paired *t-test*, *p* = 0.16; Figure 3C). Baseline EPSP area was not significantly different between fast-spiking cells in lesioned animals compared to controls (Control: 135.5 ± 15.6 mV*ms, *n* = 18; Lesion: 105.9 ± 21.9 mV*ms, *n* = 8; two-tailed *t-test*, *p* = 0.29; Figure 3C).

**Figure 4:**
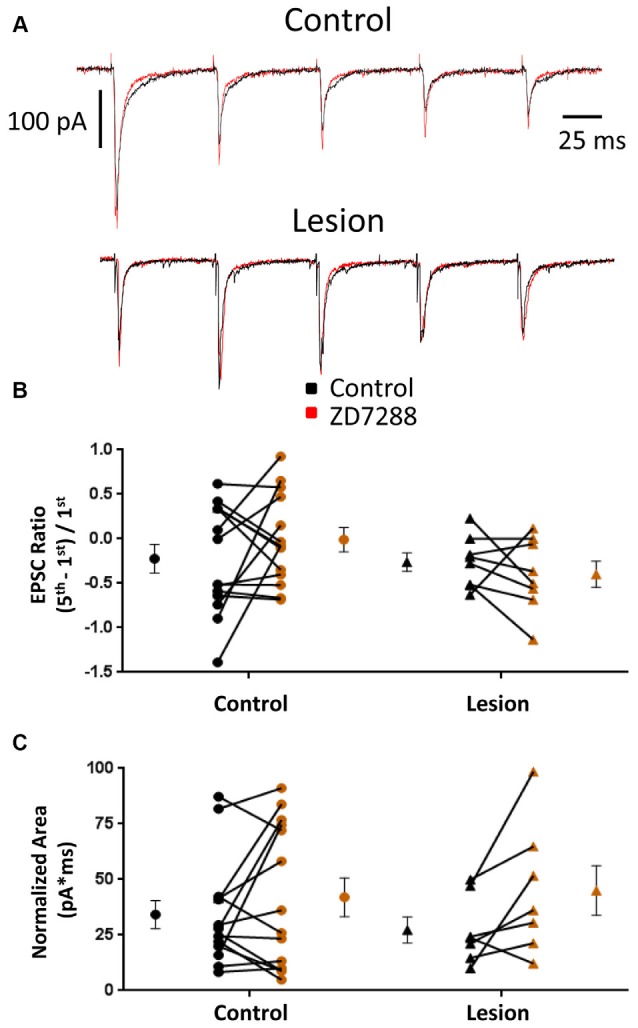
**EPSC properties in L5 fast spiking interneurons are not altered by HCN channel blockade. (A)** Example traces of EPSCs evoked at 25 Hz in interneurons before (black) and after (red) ZD7288 application in control (top) and lesioned (bottom) animals. **(B)** HCN channel inhibition did not change EPSC ratios in control or freeze lesioned animals. No difference was seen in pre-drug ratios between control and freeze lesioned rats (Control: *n* = 14, Lesion: *n* = 8). **(C)** HCN channel inhibition also had no effect on the area under the EPSC train in control or freeze lesioned rats. No difference was seen in pre-drug area between control and freeze lesioned rats (Control: *n* = 14, Lesion: *n* = 7). Error bars are mean ± SEM.

EPSCs were also examined in interneurons voltage clamped at −60 mV to minimize *I*_h_ activation (Robinson and Siegelbaum, 2003). Any effect on synaptic integration observed under these conditions would likely be mediated by presynaptic changes and not alterations intrinsic to the recorded cell. Specimen records obtained from control and lesioned groups are shown in Figure 4, upper and lower traces, respectively. Bath application of ZD7288 did not have a significant effect on initial EPSC amplitude in either the control (Control: 118.0 ± 28.9 pA, *n* = 8; ZD7288: 113.7 ± 32.6 pA, *n* = 8; two-tailed paired *t-test*, *p* = 0.71) or lesion group (Control: 60.0 ± 19.8 pA, *n* = 6; ZD7288: 42.7 ± 11.6 pA, *n* = 6; two-tailed paired *t-test*, *p* = 0.16). Under voltage-clamp conditions, evoked EPSCs did not exhibit significant summation in cells from control or lesioned animals (Figures 4A,B). HCN channel inhibition with ZD7288 did not significantly change EPSC ratios in either the control (Control: −0.22 ± 0.16, *n* = 14; ZD7288: −0.01 ± 0.14, *n* = 14; two-tailed paired *t-test*, *p* = 0.27) or lesioned groups (Figure 4B; Control: −0.26 ± 0.10, *n* = 8; ZD7288: −0.40 ± 0.15, *n* = 8; two-tailed paired *t-test*, *p* = 0.43). No difference was seen in baseline EPSC ratios between interneurons from control and lesion animals (Control: −0.22 ± 0.16, *n* = 14; Lesion: −0.26 ± 0.10, *n* = 8; two-tailed *t-test*, *p* = 0.89). ZD7288 had no significant effect on EPSC area in either the control (Control: 34.2 ± 6.4 pA*ms, *n* = 14; ZD7288: 42.0 ± 8.7 pA*ms, *n* = 14; two-tailed paired *t-test*, *p* = 0.27) or lesion group (Control: 27.3 ± 5.8 pA*ms, *n* = 7; ZD7288: 45.1 ± 11.2 pA*ms, *n* = 7; two-tailed paired *t-test*, *p* = 0.07; Figure 4C). Baseline EPSC area was also not different between groups (Control: 34.2 ± 6.4 pA*ms, *n* = 14; Lesion: 27.3 ± 5.8 pA*ms, *n* = 7; two-tailed *t-test*, *p* = 0.50). These results suggest that the observed effects of HCN channel inhibition on EPSP area in fast spiking interneurons are postsynaptically mediated.

### Alterations in Excitability in Accommodating GABAergic Interneurons in Malformed Cortex Induced by HCN Channel Inhibition

**Figure 5:**
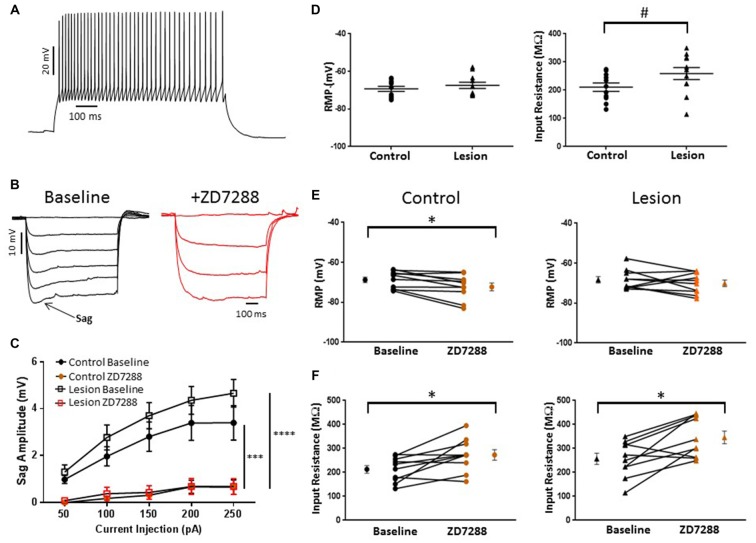
**HCN channel blockade significantly alters the intrinsic properties of accommodating interneurons in control animals. (A)** Typical spiking response from a L5 accommodating interneuron. **(B)** Typical voltage “sag” (arrow) in response to hyperpolarizing current pulses from control animals before (black trace) and after (red trace) administration of ZD7288. The first three −50 pA current steps are shown following ZD7288 application. Sag response was blocked in the presence of the HCN channel antagonist. **(C)** Quantification of voltage sag amplitudes before and after ZD7288 for control (*n* = 10, 2-way ANOVA, *p* < 0.001) and lesion (*n* = 11, 2-way ANOVA, *p* < 0.0001) groups. **(D)** RMP (left) and input resistance (right) for control and freeze lesion groups at baseline (Control: *n* = 11, Lesion: *n* = 11). **(E)** Blockade of HCN channels significantly hyperpolarized cells from control (left; *n* = 10) but not lesion groups (right; *n* = 10). **(F)** The input resistance of interneurons from both control (left; *n* = 10) and lesion groups (right; *n* = 10) was significantly increased following ZD7288 application. *p < 0.05, paired *t-test*; ^#^*p* < 0.05 *t-test*. Error bars are mean ± SEM. *** Indicates *p* < 0.01; ****p < 0.0001.

In the present study, putative Martinotti cells were identified by responses to de- and hyperpolarizing current pulses and are referred to as accommodating interneurons. A typical response of an accommodating cell in the control group to depolarizing current injection is shown in Figure 5A. Decreases in firing rate with time can be seen. Cells were further identified by their response to hyperpolarizing current pulses. A marked “sag” upon hyperpolarization, indicative of HCN channel activation, was seen (Figure 5B, baseline). Sag responses were blocked by bath application of ZD7288 (Figure 5B, +ZD7288). Figure 5C shows a plot of sag amplitude as a function of injected current for both control and lesion groups. In both groups, response amplitude was reduced at all intensities in the presence of the HCN channel inhibitor ZD7288 (Control: *n* = 10, 2-way ANOVA, *p* < 0.001; Lesion: *n* = 11, 2-way ANOVA, *p* < 0.0001). Rebound responses were also observed in these cells at the offset of hyperpolarizing current steps. Application of ZD7288 caused a significant reduction of the rebound amplitude in control and lesion groups (Control: *n* = 10, 2-way ANOVA, *p* < 0.0001; Lesion: *n* = 11, 2-way ANOVA, *p* < 0.0001). As shown in Figure 5C, sag responses increased in amplitude as a function of current injection. Responses amplitudes were not significantly different between control and lesion groups (Control: *n* = 11, Lesion: *n* = 11; 2-way ANOVA, *p* = 0.24). In control animals, significantly larger sag (Fast-spiking: *n* = 22, Accomodating: *n* = 10; 2-way ANOVA, *p* < 0.0001) and rebound responses (Fast-spiking: *n* = 10, Accomodating: *n* = 10; 2-way ANOVA, *p* < 0.0001) were observed in accommodating cells compared to fast-spiking cells (data not shown). The resting membrane potentials of accommodating interneurons in the control and lesion groups were not significantly different (Control: −69.4 ± 1.4 mV, *n* = 11; Lesion: −67.4 ± 1.7 mV, *n* = 11; two-tailed *t-test*, *p* = 0.38; Figure 5D, left). However, the input resistance of accommodating cells was significantly higher in the lesion group compared to controls (Control: 210.7 ± 14.7 MΩ, *n* = 11; Lesion: 258.9 ± 21.4 MΩ, *n* = 11; two-tailed *t-test*, *p* < 0.05; Figure 5D, right). The effect of HCN channel inhibition on resting membrane potential and input resistance was examined in both groups. As shown in Figure 5E, bath application of ZD7288 was associated with a small but significant hyperpolarization in the control group (Control: −68.8 ± 1.3 mV, *n* = 10; ZD7288: −72.3 ± 1.9 mV, *n* = 10; paired *t-test*, *p* < 0.01; Figure 5E, left) whereas RMP was not significantly changed in the lesion group (Control: −68.3 ± 1.6 mV, *n* = 10; ZD7288: −70.1 ± 1.6 mV, *n* = 10; paired *t-test*, *p* = 0.16; Figure 5E, right). Input resistance was significantly increased in both control (Control: 212.6 ± 16.1 MΩ, *n* = 10; ZD7288: 272.7 ± 21.5 MΩ, *n* = 10; paired *t-test*, *p* < 0.01) and lesion groups (Control: 256.6 ± 23.5 MΩ, *n* = 10; ZD7288: 346.0 ± 26.2 MΩ, *n* = 10; paired *t-test*, *p* < 0.01; Figure 5F, left and right, respectively).

### Synaptic Integration in L5 Accommodating Interneurons

**Figure 6:**
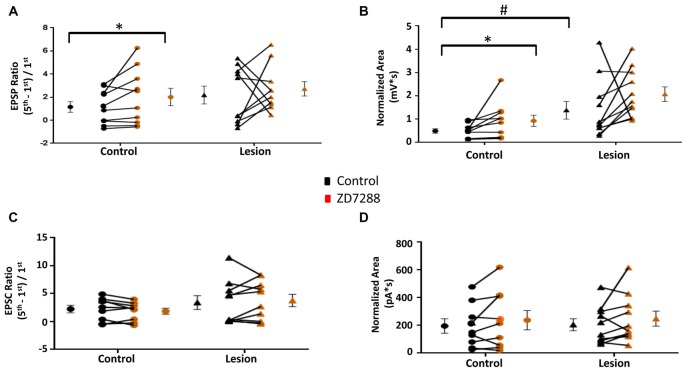
**EPSP ratios in L5 accommodating interneurons are increased in control but not freeze lesioned animals following HCN blockade. (A)** HCN channel inhibition increased amplitude ratios in control but not freeze lesioned animals. No difference was seen in pre-drug ratios between control and lesion groups (Control: *n* = 10, Lesion: *n* = 10). **(B)** EPSP area was significantly increased following ZD7288 application in control (left) but not lesion groups. Pre-drug area was significantly increased in freeze lesioned rats compared to controls (Control: *n* = 10, Lesion: *n* = 11). **(C,D)** Blockade of HCN channels did not alter EPSC ratios (C; Control: *n* = 9, Lesion: *n* = 11) or EPSC areas (D; Control: *n* = 9, Lesion: *n* = 10) in control or lesioned animals. *p < 0.05, paired *t-test*; ^#^*p* < 0.05 *t-test*. Error bars are mean ± SEM.

The above results indicate that L5 accommodating interneurons have HCN channel mediated sag responses and changes in RMP and input resistance consistent with the presence of an *I*_h_ current. We therefore examined the effect of HCN channel inhibition on evoked EPSPs and EPSCs in these interneurons. Trains of 25 Hz stimulation in interneurons from control animals produced EPSP ratios which were significantly increased by ZD7288 in the control (Control: 1.15 ± 0.46, *n* = 10; ZD7288: 2.01 ± 0.75, *n* = 10; paired *t*-test, *p* < 0.05; Figure 6A left) but not the lesion group (Control: 2.18 ± 0.77, *n* = 10; ZD7288: 2.72 ± 0.62, *n* = 10; paired *t-test*, *p* = 0.30; Figure 6A, right). ZD7288 did not increase the amplitude of the initial evoked EPSP in interneurons from control (Control: 1.48 ± 0.24 mV, *n* = 10; ZD7288: 1.29 ± 0.32 mV, *n* = 10; paired *t-test*, *p* = 0.27) or lesion animals (Control: 1.94 ± 0.88 mV, *n* = 11; Lesion: 0.63 ± 0.15 mV, *n* = 11; paired *t-test*, *p* = 0.08). Analysis of EPSP areas showed that ZD7288 increased the area significantly in the control group (Control: 0.49 ± 0.09 mV*s, *n* = 10; ZD7288: 0.93 ± 0.24 mV*s, *n* = 10; paired *t-test*, *p* < 0.05; Figure 6B left), but not the lesion group (Control: 1.38 ± 0.38 mV*s, *n* = 11; ZD7288: 2.07 ± 0.31 mV*s, *n* = 11; paired *t-test*, *p* = 0.09; Figure 6B, right). Prior to ZD7288 treatment, the control group had a significantly smaller EPSP area compared to the lesion group (Control: 0.49 ± 0.09 mV*s, *n* = 10; Lesion: 1.38 ± 0.38 mV*s, *n* = 11; two-tailed *t-test*, *p* < 0.05).

In another series of experiments, EPSCs were evoked in accommodating interneurons while cells were voltage clamped at −60 mV to minimize *I*_h_ activation (Robinson and Siegelbaum, 2003). Bath application of ZD7288 did not have a significant effect on initial EPSC amplitude in the control (Control: 24.1 ± 6.8 pA, *n* = 9; ZD7288: 23.6 ± 7.4 pA, *n* = 9; two-tailed paired *t-test*, *p* = 0.90) or lesion group (Control: 38.1 ± 15.4 pA, *n* = 11; Lesion: 25.4 ± 8.2 pA, *n* = 11; two-tailed paired *t-test*, *p* = 0.20). Under voltage-clamp conditions, evoked EPSC ratios were variable in interneurons from both control and lesion animals (Figure 6C). HCN channel inhibition with ZD7288 did not significantly change EPSC ratios in either the control (Control: 2.25 ± 0.68, *n* = 9; ZD7288: 1.85 ± 0.54, *n* = 9; two-tailed paired *t-test*, *p* = 0.20) or lesion groups (Control: 3.41 ± 1.23, *n* = 10; ZD7288: 3.78 ± 1.11, *n* = 10; two-tailed paired *t-test*, *p* = 0.54; Figure 6C). There was also no significant change in the EPSC area in either the control (Baseline: 159.9 ± 51.7 pA*s, *n* = 9; ZD7288: 237.9 ± 69.1 pA*s, *n* = 9; paired *t-test*, *p* = 0.17) or lesion group (Control: 205.4 ± 42.7 pA*s, *n* = 10; ZD7288: 249.6 ± 54.6 pA*s, *n* = 10; paired *t-test* *p* = 0.23; Figure 6D). These results suggest that, as with fast-spiking interneurons, the effects of HCN channel inhibition on EPSP integration are mediated postsynaptically.

## Discussion

In this study, we examined the role of HCN channels in regulating intrinsic and synaptic excitability of two classes of L5 interneurons in rat neocortex. In addition, we assessed whether HCN channel modulation of excitability was changed in lesioned animals. Our results indicate that inhibition of HCN channels differentially affects fast spiking (presumed basket) cells and accommodating (presumed SOM positive) neurons and that properties of these GABAergic interneurons are altered in malformed cortex.

### GABAergic Interneurons and HCN Channels

Immunocytochemical studies have documented HCN channel expression in the dendrites of neocortical and hippocampal pyramidal cells with a pronounced increase in density as a function of distance from the soma (Lörincz et al., 2002; Notomi and Shigemoto, 2004). However, this pattern is not invariant. Cerebellar Purkinje cells exhibit a uniform HCN dendritic density (Angelo et al., 2007) whereas a subpopulation of CA1 neurons, termed pyramidal-like principal neurons, displays a reversed somatodendritic gradient (Bullis et al., 2007). In both cases, HCN channel inhibition still altered EPSP summation. Hippocampal basket cell axons and axon terminals show HCN immunoreactivity (Notomi and Shigemoto, 2004). HCN channel inhibition increased the threshold for evoking antidromic action potentials in these cells and decreased miniature IPSC frequency (Aponte et al., 2006). Although neocortical interneurons do not typically stain for HCN channels (Lörincz et al., 2002), functional studies of HCN channels suggest the presence of HCN channels in certain subclasses of neocortical GABAergic interneurons (Ma et al., 2006). Although the channel location is still undetermined, our results indicate that HCN channel inhibition alters EPSP ratios in accommodating, but not fast spiking, interneurons.

Three main subtypes of cortical GABAergic interneurons have been extensively characterized electrophysiologically. These are the SOM-positive Martinotti cells, the neurogliaform (NGF) cells and the fast-spiking PV-expressing basket cells. SOM-positive cells display ascending axon collaterals which reach L1 and ramify extensively (Kawaguchi and Kubota, 1997; Wang et al., 2004). They can be sub-classified as regular spiking or burst firing (Wang et al., 2004; Uematsu et al., 2008), mediate frequency-dependent disynaptic inhibition and target oblique, apical and tuft dendrites of L5 pyramidal cells (Silberberg and Markram, 2007; Berger et al., 2010). HCN channel-mediated sag responses have been observed in SOM-positive cells (Ma et al., 2006). Facilitating EPSPs from pyramidal cells to SOM-positive cells were slightly reduced in the presence of ZD7288 to block HCN channels (Berger et al., 2010). Neurogliaform cells display a late-spiking electrophysiological phenotype (Kawaguchi, 1995; Tamás et al., 2003; Povysheva et al., 2007), have a large number of primary dendrites emanating from a small round soma (Karagiannis et al., 2009; Wozny and Williams, 2011) and produce both GABA_A_ and GABA_B_ receptor mediated responses in pyramidal cells (Simon et al., 2005). Basket cells can sustain high firing rates without showing accommodation and form dense and unspecific connections to pyramidal cells (Kawaguchi and Kubota, 1997). Driving basket cells induces gamma rhythm activity (Cardin et al., 2009) and entrains excitatory neurons (Fries et al., 2001; Hasenstaub et al., 2005). The effect of HCN channel inhibition on synaptic integration in SOM- and PV-positive interneurons has not been extensively examined.

### HCN Channels and GABAergic Interneuron Excitability

#### Fast Spiking Interneurons

The RMP and input resistance of fast-spiking interneurons in control and lesioned groups were not significantly different. HCN channel inhibition did not significantly affect the RMP in either group whereas input resistance was increased in both. HCN channel mediated sag responses elicited by hyperpolarizing current pulses were small and had an atypical time course in the control group. In the lesion group, sag responses were absent or significantly reduced. Responses in both control and lesion groups were significantly reduced by ZD7288. Overall, this complex pattern of changes is consistent with the presence of HCN channels capable of influencing input resistance but not RMP. The location of these channels is unclear as is the factors responsible for the unusual time course of sag responses.

In the fast spiking cells, inhibition of HCN channels with ZD7288 did not significantly affect singly-evoked EPSPs in interneurons from control or lesioned groups. EPSP area during short trains of stimulation was increased in the control group after pharmacological inhibition of HCN channels whereas increased area was not observed in the lesion group. This is consistent with a loss of HCN modulation of EPSPs in the lesion group. When neurons were voltage clamped at −60 mV, no effect of ZD7288 on EPSC area was observed in either the control or lesion group, suggesting that HCN channels modulate excitatory inputs onto L5 basket cells via a postsynaptic mechanism. In the present study, we demonstrated that L5 fast-spiking cells had small atypical responses to hyperpolarizing current pulses, implying the existence of small, possible remote HCN-mediated *I*_h_ currents. We have previously shown that HCN mediated responses are reduced in L5 pyramidal neurons in freeze lesioned rats (Albertson et al., 2011). The present results indicate that HCN channels may be similarly reduced in nearby fast spiking interneurons, suggesting that loss of HCN channels may occur in multiple cell types in malformed cortex.

#### Accommodating Interneurons

Interneurons with accommodating firing patterns are presumptive SOM-positive cells. The RMP of accommodating interneurons in control and lesioned groups were not significantly different whereas input resistance was significantly higher in the lesion group. RMP was hyperpolarized in the presence of ZD7288 by a small but significant amount in the control but not the lesion group. Input resistance was significantly increased in by groups by HCN channel inhibition. In keeping with previous studies (Ma et al., 2006), sag responses in these cells were larger in amplitude and had a more characteristic time course compared to fast spiking interneurons. It is also becoming clear that accommodating cells are heterogeneous and changes may have occurred in subpopulations. These complex changes are nonetheless suggestive of HCN modulation of membrane excitability in these cells.

HCN channel inhibition increased EPSP ratios and area in controls but not the lesioned group. This suggests that HCN channels play a significant role in controlling synaptic excitability of accommodating interneurons, a role that is disrupted in malformed cortex. The functional significance of this for seizures needs to be established. The differential effects on EPSP ratios and sag responses in the lesion group could potentially be due to the observed increases in input resistance or differential effects on somatic and dendritic HCN channels.

### Complexity of Circuit Modulation by HCN Channels

Defining the role of *I*_h_ in individual circuit elements including interneurons is essential for understanding how the loss of HCN channels alters network excitability. Imbalance of excitation and inhibition contributes to aberrant network activity (Yizhar et al., 2011) as well as epileptiform hyperexcitability (Gutnick et al., 1982). Loss of interneurons has been implicated as a potential contributor to hyperexcitability in a developmental epilepsy model (Gill et al., 2010) and in the irradiation model of cortical dysplasia (Xiang et al., 2006; Zhou et al., 2009). There is also evidence of dysfunctional inhibition in human cortical dysplasia (André et al., 2010). Fast spiking basket cells in irradiated cortex show decreases in spontaneous and miniature EPSCs. Since fast-spiking basket cells are one of the most abundant subtypes of interneuron in the neocortex (Rudy et al., 2011), loss of excitation in these neurons could lead to increased network excitability. Impaired HCN channel inhibitory modulation of EPSP summation in lesioned animals could actually serve as a protective mechanism and help preserve the excitation-inhibition balance. While the effect of HCN channel loss on fast spiking cell excitation was not as large as that observed previously in pyramidal neurons (Shin et al., 2008; Huang et al., 2009; Albertson et al., 2011) the changes in accommodating cells was significant. These data suggest that loss of HCN channels may differentially influence excitatory and inhibitory network elements, potentially contributing to an imbalance of excitation and inhibition.

One alternative mechanism is increased presynaptic release of glutamate following HCN channel inhibition. HCN channels have previously been implicated in the control of GABA release. In the cerebellum, basket cell terminals show immunogold labeling for HCN channels (Luján et al., 2005) whereas HCN channel inhibition decreases the rate of spontaneous inhibitory currents in amygdala (Park et al., 2011) and hippocampus (Peng et al., 2010). When interneurons were voltage clamped at −60 mV, EPSCs in response to short trains of stimuli were not changed following HCN channel inhibition. Under these conditions, interneurons were clamped at a potential which likely precluded HCN channel opening (Berger et al., 2001). These data suggest that loss of HCN channels does not change release at these synapse, in agreement with previous work suggesting that presynaptic HCN channels are expressed in a synapse specific, not global, manner (Huang et al., 2011). Therefore, the changes in EPSPs seen after HCN channel inhibition are likely due to a postsynaptic mechanism.

In summary, HCN channel inhibition alters intrinsic and synaptic properties of GABAergic interneurons. We observed an increase in EPSP ratios in accommodating interneurons following HCN channel inhibition suggesting that GABA release could be increased. However, the increase was not as robust as that observed previously in pyramidal cells. The functional consequences of HCN loss may differ between excitatory and inhibitory cell types. These data suggest that while HCN channels may serve a similar dendritic filtering role in some interneurons, HCN channel influence in pyramidal neurons is greater. We also did not observe any evidence of presynaptic HCN channels at excitatory synapses onto fast spiking L5 interneurons.

## Author Contributions

AJA, ASB and JJH designed the research and wrote the manuscript. AJA and ASB conducted experiments and analyzed the data.

## Conflict of Interest Statement

The authors declare that the research was conducted in the absence of any commercial or financial relationships that could be construed as a potential conflict of interest.
